# Systematic review of the evidence on the effectiveness of sexual and reproductive health interventions in humanitarian crises

**DOI:** 10.1136/bmjopen-2015-008226

**Published:** 2015-12-18

**Authors:** Emily Warren, Nathan Post, Mazeda Hossain, Karl Blanchet, Bayard Roberts

**Affiliations:** 1 Faculty of Epidemiology and Population Health, Department of Infectious Disease Epidemiology, London School of Hygiene & Tropical Medicine, London, UK; 2 Faculty of Medical Sciences, Newcastle University, Newcastle upon Tyne, UK; 3 Faculty of Public Health Policy, Department of Global Health and Development, London School of Hygiene & Tropical Medicine, London, UK; 4 Faculty of Infectious and Tropical Diseases, Department of Clinical Research, London School of Hygiene & Tropical Medicine, London, UK; 5 Faculty of Public Health Policy, Department of Health Services Research and Policy, London School of Hygiene & Tropical Medicine, London, UK

**Keywords:** REPRODUCTIVE MEDICINE, SEXUAL MEDICINE, HEALTH SERVICES ADMINISTRATION & MANAGEMENT, PUBLIC HEALTH

## Abstract

**Objectives:**

This systematic review aims to evaluate evidence on the effectiveness of sexual and reproductive health (SRH) interventions delivered in humanitarian crises.

**Setting:**

Crisis affected low-income or middle-income countries.

**Participants:**

Crisis-affected populations in low-income or middle-income countries.

**Method:**

Peer-reviewed and grey literature sources were systematically searched for relevant papers detailing interventions from 1 January 1980 until the search date on 30 April 2013. Data from included studies were then extracted, and the papers’ quality evaluated using criteria based on modified STROBE and CONSORT checklists.

**Primary and secondary outcome measures:**

Primary outcomes include, but are not limited to, changes in morbidity, mortality, sexually transmitted infection (STI) diagnosis or gender-based violence. Secondary outcomes include, but are not limited to, reported condom use or skilled attendance at birth. Primary outputs include, but are not limited to, condoms distributed or education courses taught.

**Results:**

Of 7149 returned citations, 15 studies met the inclusion criteria. Only one randomised controlled trial was identified. The remaining observational studies were of moderate quality, demonstrating limited use of controls and inadequate attempts to address bias. Evidence of effectiveness was available for the following interventions: impregnated bed nets for pregnant women, subsidised refugee healthcare, female community health workers, and tiered community reproductive health services.

**Conclusions:**

The limited evidence base for SRH interventions highlights the need for improved research on the effectiveness of public health interventions in humanitarian crises. While interventions proven efficacious in stable settings are being used in humanitarian efforts, more evidence is required to demonstrate the effectiveness of delivering and scaling-up such interventions in humanitarian crises.

**Strengths and limitations of this study**

- Broad search strategy including peer-reviewed and grey literature.
- Rigorous assessment of the quality and strength of evidence.
- Only quantitative studies are included.
- No meta-analysis was possible so a narrative summary of the results is provided.

## Introduction

Humanitarian crises can increase vulnerability to poor sexual and reproductive health (SRH) outcomes among affected populations due to reduced access to SRH services and supplies, damaged health facilities, depleted human resources, increased exposure to sexual violence, and increased impoverishment and related risk-taking.^1–3^ As a result, there is evidence of high unmet needs and insufficient investments for SRH services during humanitarian crises.^4–6^

The responsibility of humanitarian actors to respond to the SRH needs of those affected by conflict and natural disasters has been increasingly recognised over the past two decades. Following the 1994 International Conference on Population Development in Cairo,^7^ ^8^ where the sexual and reproductive needs of those affected by humanitarian crises were explicitly acknowledged, the Inter-Agency Working Group for Reproductive Health in Refugee Settings (IAWG) was formed and in 1999 they produced, along with the WHO, the Inter-Agency Field Manual on Reproductive Health in Humanitarian Settings.^9^ The manual provides guidance on the use of the Minimum Initial Service Package (MISP) in order to meet the most pressing needs related to family planning, HIV/AIDS, and sexually transmitted infections (STIs) among conflict and disaster-affected populations.^9^ While the evidence on the effectiveness of SRH interventions in stable resource-constrained settings is extensive^10^ the evidence-base for delivering SRH interventions in humanitarian settings is less well documented.

It is important to gauge the evidence on the effectiveness of SRH interventions specifically in humanitarian contexts because the ability to deliver SRH services in such contexts may be different due to issues such as insecurity, limited financial and human resources, and population mobility.

To address these evidence gaps, the aim of this systemic review is to evaluate the evidence on the effectiveness of SRH interventions delivered in humanitarian crises. This study is part of a larger project evaluating the evidence on the effectiveness of interventions on a range of health outcomes in humanitarian settings.^11^

## Methods

This systematic literature review adheres to the Preferred Reporting Items for Systematic Reviews and Meta-Analyses (PRISMA) statement.^12^ The inclusion and exclusion criteria are detailed in table 1.

**Table 1:** Inclusion and exclusion criteria

Category	Included	Excluded
Population of interest	Crisis-affected populations receiving humanitarian assistance or aid in low-income or middle-income countries (as defined by World Bank, 2012): including refugees and internally displaced persons	Studies on preparedness or resilience if not linked to an intervention which evaluates effectiveness
Health outcomes or outputs	Primary outcomes (changes in morbidity, mortality, STI diagnosis, gender-based violence), secondary outcomes (reported condom use, skilled attendance at birth) and primary outputs (condoms distributed, education courses taught)	Studies which do not quantify health outcomes or outputs
Intervention	Any health-related intervention seeking to improve SRH outcomes	
Comparison	Measurements taken before or after an intervention or with use of a control group not receiving an intervention	
Phase of humanitarian crises	Studies conducted during the acute, chronic and early recovery phases of a humanitarian crisis	Studies conducted before or after a crisis has stabilised
Study types and designs	All quantitative study designs measuring change in health outcomes over time	Qualitative studies and quantitative studies which do not measure changes in health outcomes or outputs
Publication date	1 January 1980 to 30 April 2013	
Language	English, French	Other languages

SRH, sexual reproductive health; STI, sexually transmitted infection.

Search terms for sexual and reproductive health were based on the standardised definition from the International Conference on Population and Development in 1994. SRH refers to “the constellation of methods, techniques and services that contribute to reproductive health and well-being by preventing and solving reproductive health problems.” International guidelines on reproductive health in conflict-affected situations include reproductive activities on family planning, HIV/AIDS and sexually transmitted diseases, maternal and newborn health, and sexual and gender-based violence.^13^ ^14^

For the purposes of this review a humanitarian crisis is defined as a serious disruption of the functioning of a community or a society causing widespread human, material, economic or environmental losses which exceed the ability of the affected community or society to cope using its own resources, necessitating a request to national or international level for external assistance. The disaster situation may either be man-made (eg, armed conflict) or a natural phenomenon (eg, drought). Although crises can affect all countries, only studies from low-income or middle-income countries were included in this study. The majority of humanitarian crises occur in these countries, and the resources available to address them are very different in high-income countries. For these reasons, the study team included only papers from low-income and middle-income countries.

The specific search terms (see online supplementary annex 1) were generated by the authors and then supplemented by searching for other search strategies used in previous systematic reviews on similar topics.^15^ ^16^ A trained information science and Cochrane review specialist was also consulted with to ensure proper literature searching syntax and strategy were used.

Peer-reviewed journals and grey literature reports were included. We searched for peer-review published literature across three databases: EMBASE, Global Health and MEDLINE. Grey literature was searched using online sources: R4D, Reproductive Health Response in Crisis Consortium (RHRC), Medecins Sans Frontiers (MSF) Field Research, UNFPA, RAISE Initiative, IAWG, Save the Children, The International Rescue Committee (IRC), CARE, International Committee of the Red Cross (ICRC), International Planned Parenthood Federation (IPPF), AIDS Alliance and Marie Stopes International (MSI). Searches were complemented by screening the reference lists of papers for potentially relevant studies. Experts on SRH service delivery and research were also consulted to identify any additional research not found during the systematic search.

**Figure 1:**
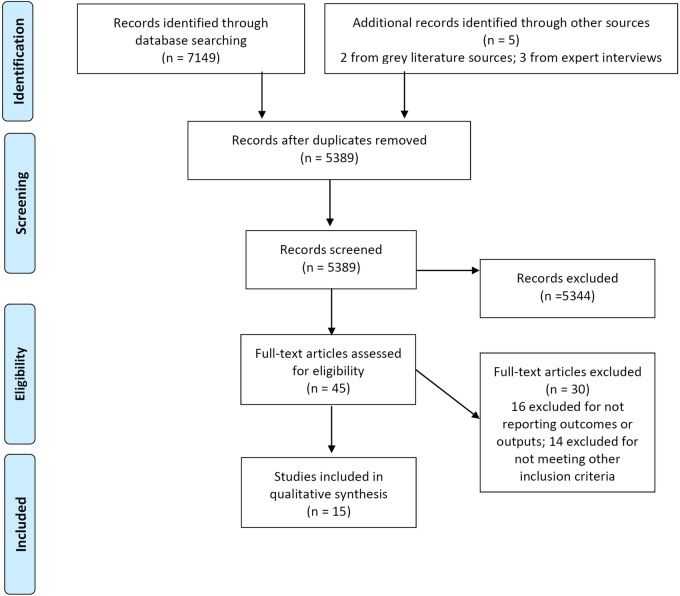
Adapted the Preferred Reporting Items for Systematic Reviews and Meta-Analyses (PRISMA) flow chart.

All returned citations were downloaded into an Endnote library and a standard data-screening process was applied (figure 1). Primary and secondary outcomes and primary outputs of interest used for inclusion were derived from the IAWG field manual.^14^ This is an established and widely used manual for SRH in crisis-affected settings, and was selected based on discussion with SRH experts. Those listed in table 1 represent the actual outcomes and outputs ultimately identified in the included literature. Data from the final selected studies were then extracted into an Excel database, with the data extraction fields including study design and methods, research setting, health outcomes and intervention descriptions. First round data screening and extraction were independently conducted by EW and MH in duplicate. Second round detailed data extraction was conducted independently and in duplicate by EW and NP.

Owing to the heterogeneity of study outcomes, interventions and methods, a descriptive analysis was chosen over a meta-analysis. The findings were synthesised by main SRH outcomes (family planning; prevention, treatment, care for STIs including HIV/AIDS; maternal, newborn and child health, including obstetric care; and health system strengthening). These themes were developed iteratively after thematic analysis of the studies’ stated aims and primary reported health outcome of interest.

The quality of reporting in the included studies were assessed using the Strengthening the Reporting of Observational Studies in Epidemiology (STROBE) and Consolidated Standards of Reporting Trials (CONSORT) checklists, which are commonly used for reporting the quality of observational and trial studies.^17^ ^18^ The STROBE and CONSORT checklists include measures regarding the reporting of participant selection, variables, data sources, bias, descriptive and outcome data, interpretation, and generalisability, among others. In order to further explore the quality of reporting, papers were awarded one point for reporting each of the items on the relevant checklist. These points when totalled formed the numerator of a proportional score, with the denominator the total number of possible relevant checklist items which varied slightly by study type. The quality assessment was conducted by EW and NP who each independently evaluated the quality of all included studies and discussed each discrepancy until consensus was reached. For this systematic review, the study team determined a priori that papers with a score of <33% were considered low reporting quality, moderate quality if 34–66%, and high quality if >67%. Particular features consistently showing low-quality scores, were also identified and discussed in further detail.

## Results

In total 7149 citations were returned from peer-reviewed sources, two from grey literature libraries and three from expert recommendations. After screening, 15 studies meeting our inclusion criteria were selected (see online supplementary table S2 and annex 2). No studies describing interventions aimed exclusively at responding to or preventing gender-based violence in crisis settings were identified. One intervention described the inclusion of gender-based violence topics but did not include any measurements to assess its influence.^19^ Studies which examined mental health outcomes for survivors of gender-based violence (GBV) are described elsewhere.^11^

The 15 SRH studies were conducted in 10 different countries with populations affected by conflict. No study conducted in a natural disaster setting was identified. Between 1990 and 1999, three papers meeting our criteria were published. Between 2000 and 2009, this figure increased to seven, and between 2010 and 2013, five additional papers were found.

## Family planning

Two studies were identified whose primary aim was to examine family planning in conflict-affected settings in northern Uganda and Pakistan.^20^ ^21^ Both indicated that when family planning services are available and accessible, women's use of modern family planning increased.^20^ ^21^ In northern Uganda, a case-series study evaluating a mobile health outreach and public health centre strengthening programme recorded an increase in current use of modern family planning between 2007 and 2010 from 7.1% to 22.6% (adjusted OR (AOR) 3.34; 95% CI 2.27 to 4.92).^20^ Current use of long-acting or permanent methods of family planning also increased from 1.2% to 9.8% over the same period (OR 9.45; 95% CI 3.98 to 22.42). In the second study, female Afghan refugees in Pakistan receiving subsidised healthcare reported more than double the use of modern family planning methods compared to those who did not receive subsidised healthcare (54% vs 25%; p=0.000) and they were six times more likely to discuss the number of children they wanted with their partners (p=0.000).^21^ Despite these increases, the cross-sectional study design precludes attributing healthcare subsidies to increases in the uptake of family planning services.

Three other publications also reported family planning as secondary health outcomes of interest.^19^ ^22^ ^23^ Viswanathan,^23^ using a case–control study and Mullany,^22^ using a case-series study observed an increased use in modern contraception following the implementation of community-based health worker interventions in Afghanistan (OR 1.61; 95% CI 1.12 to 2.15) and among internally displaces populations in eastern Burma (23.9% to 45.0%; prevalence rate ratio (PRR) 1.88; 95% CI 1.63 to 2.17). McGinn reported that following a literacy intervention with Sierra Leonean and Liberian camp-based refugees in Guinea, 40% of women started using modern family planning methods after being exposed to an educational programme with a focus on safe motherhood, family planning, STIs, HIV/AIDS and gender-based violence.^19^

## Prevention, treatment and care for STIs, including HIV/AIDS

Four studies were identified that aimed to improve prevention, treatment, and care for STIs. Two were education based^24^ ^25^ and two focused on clinical management.^26^ ^27^

Woodward *et al*^25^ assessed the impact of refugee-led peer education on refugees living in Guinea through a cross-sectional evaluation. Refugees exposed to peer education had over double the odds of having heard about HIV (OR 2.19; 95% CI 1.58 to 3.05) compared to unexposed individuals and were more likely to identify both correct and erroneous HIV transmission pathways, including only having one partner (OR 3.24; 95% CI 1.62 to 6.44 (adjusted for gender)), using condoms (OR 1.91; unadjusted 95% CI 1.15 to 3.16), and sharing food with HIV-positive people (OR 1.52; unadjusted 95% CI 1.10 to 2.10). Participants also had 2.5 times greater odds than non-participants of reporting increased HIV avoidant behaviours (72% vs 58%; OR 2.49, 95% CI 1.52 to 4.08 (adjusted for gender and age of sexual debut age)).^25^

A separate educational intervention targeting military and female commercial sex worker (CSW) communities in Port Loko, Sierra Leone evaluated by a case-series design, found promising trends in improved knowledge, attitude and behavioural changes (all reported p values <0.01).^24^ The study, however, did not include detailed information on the intervention contents and process. The CSW's knowledge of three or more ways of avoiding HIV rose from 5.0% to 70.3% between baseline and endline over a 2-year period. Knowledge of two or more STI symptoms also rose from 46.8% to 88.1% (p<0.01). Behavioural indicators, such as reported condom use at last sexual intercourse, also rose between baseline and endline surveys from 38% to 68%, although no significance statistics for this indicator were presented.^24^

Working in Tanzania with Rwandan refugees, Mayaud *et al*^26^ documented an early humanitarian response in a high HIV prevalence setting. The intervention used health promoters and mass-distributed education materials, peer educators among bar and brothel workers, condom distribution, an STI intervention including training and supervising health workers, a syndromic approach for STI case management, medicine provision, and antenatal syphilis screening. During the 18-month intervention period, 120 information and education campaigns were organised and monitoring data estimated 230 000 sexually active people were reached and 1.5 million condoms were distributed throughout the camp. More than 11 000 syndromically identified patients were treated in the first year, growing to 18 000 at the end of programme, showing an increase from the 20 cases/week counted at baseline. The study authors noted that sexual behaviours were not necessarily safer as there was an increase in paid and transaction sex, and that during the intervention period, levels of sexual violence increased. The evaluation design did not allow for a causal association to be made as the findings were primarily descriptive and no significance testing was reported.

A fourth study by Reid *et al*^27^ provided intervention details and a descriptive comparison of a Médecins Sans Frontières (MSF) programme implemented in 2008 in response to election-related violence in Kenya. During the political unrest, initiation of highly active antiretroviral therapy (HAART) was temporarily halted. An intervention to ensure continuity of care was implemented where patients who required HIV treatment were given personalised clinical and treatment summaries with the names and dosage of medications. These summaries could then be used to ensure continuity of care in other clinics if MSF clinics were unable to open due to insecurity. A comparison of service record data from January to March in 2008 to the same time period in 2007, found more than twice the number of treatment interruptions during the conflict than 1 year before (13.1% compared to 6.4%, no significance testing). However, by February 2008, the difference had shrunk to 0.5% and by March there were an equal percentage of delayed appointments. Lost to follow-up was similarly controlled by March 2008. Among those enduring treatment interruptions, slightly more (36%) did not miss any pills than did (31%). However, no significance tests were reported.

## Maternal, newborn and child health, including obstetric care

Nine studies evaluated interventions focused on improving maternal, newborn and child health outcomes. One double-blind randomised control trial evaluated the efficacy of permethrin-treated bed nets compared with untreated nets or no bed net.^28^ The incidence of malaria and anaemia in pregnant women was assessed. Researchers found that the geometric mean parasite density was significantly lower in women assigned to the treated bed nets (517; 95% CI 286 to 932/mm^3^) than those who received no or non-treated nets (1096; 95% CI 726 to 1655/mm^3^; p=0.049, adjusted for gravidity).^28^ Treated bed nets halved the risk of subsequent anaemia requiring treatment compared to those randomised to receive no net (95% CI 1.2 to 2.4) and had a 1.6-fold risk reduction compared to those using a non-treated net (95% CI 1.1 to 2.4).

Three studies focused on increased community education, involvement, and birth preparedness and planning.^19^ ^29^ ^30^ The Reproductive Health Literacy Project in Guinea was carried out with Sierra Leonean and Liberian women living in refugee camps, evaluating the impact of participatory adult literacy education on improving health knowledge and health-seeking behaviour in the camp.^19^ A retrospective cross-sectional evaluation found that 40% of women had not used a modern family planning method prior to the health literacy course and 24% reported using a condom at their last sexual encounter. In total 92% of women who became pregnant since exposure to the intervention attended at least three of the four recommended antenatal visits. The study also examined changes in ‘boldness’ (understood as empowerment). Before the health literacy course, 32% of women considered themselves ‘more bold’ than other women but after the course 81% did (p=<0.001). Women who identified themselves as ‘more bold’ were more likely to use family planning (51% vs 36%, p<0.01) and a condom at last sex (26% vs 14%, p<0.05) than women who did not consider themselves ‘more bold’.^19^ However, the study is considerably weakened by the fact that it did not use baseline data. Participants were instead asked after the intervention to describe their knowledge and behaviours before and after the literacy course, which along with other weaknesses in the study's execution makes the conclusion susceptible to numerous biases and limits its ability to attribute a direct impact on changing associated SRH-related outcomes.

Purdin *et al* used an ecological study design to evaluate a programme with Afghan refugees in Pakistan which aimed to reduce maternal and neonatal mortality by increasing the use of emergency obstetric care through improving community knowledge about skilled delivery and obstetric danger signs. They observed that the maternal mortality ratio dropped from 291 to 102 per 100 000 live births from the first to the fifth year of the programme (95% CI 181 to 400). Neonatal mortality also decreased from 25 to 20.7 per 1000 births from the first to the seventh year, however no CIs were reported.^30^ However, the study uses routine observational data and so the authors’ attribution of the change in outcomes to the intervention should be treated with considerable caution given the study design cannot demonstrate causation and there is high probability of confounding.

McPherson *et al* evaluated a birth-preparedness package in Nepal using a case-series design. The intervention was a multilevel community education and healthcare response, seeking to reduce maternal and neonatal mortality and increase use of health services. Comparing endline to baseline surveys among mothers of infants, health behaviours improved—including not putting anything on the umbilical cord, wrapping the baby immediately after birth and delaying bathing (p=0.000). Attending at least one antenatal visit, making financial and transport preparations also improved (p=0.000). Breastfeeding within an hour of birth was not statistically improved (p=0.06). Of the seven chosen indicators, only the use of a skilled attendant at birth remained unchanged (p=0.55).^29^

Five studies attempted to improve maternal and neonatal health through system strengthening and capacity building.^22^ ^23^ ^31–33^ One study using a cohort design from Bo, Sierra Leone, examined if a transportation and referral system could be implemented to reduce delays in accessing emergency obstetric care from a referral hospital.^33^ In addition to improving the physical structures of the hospital, providing drugs, staff trainings and leading a community sensitisation campaign aimed at early recognition and care-seeking for obstetric emergencies, a transportation and radio communication referral system to bring women from remote areas into the hospital was established. During the intervention period of 16 months, the case fatality rate (CFR) (maternal deaths/obstetric complications) dropped from 20% to 10%, regardless of whether the patient was brought to hospital using the referral and transportation intervention or not. Utilisation of emergency obstetric care doubled, some of which may be attributable to improved service and community recognition of maternal danger. The decreased CFR could also be caused by shortened delays in seeking care. However, because the CFR decreased for all women regardless of how they came to the hospital, caution should be used before assuming causation of the referral system—particularly given the study design.

An initiative in Makeni, Sierra Leone, assessed the impact of numerous hospital-based improvements including hiring a new medical officer with training in obstetrics and a second medical officer who would receive obstetric training.^31^ Midwives and nurses were also trained and underwent refresher courses in recognising and responding to obstetric complications. An unused operating theatre was renovated, an electricity generator installed, and drugs and supplies were acquired and paid for using a revolving fund. Policies were also implemented which required treatment before payment, and community outreach workers were trained to refer women with complications to the hospital. Maternal CFR dropped from 32% in 1990 to 4% in 1994, while the number of patients increased from 31 in 1990 to 89 in 1994, although no CIs or other statistics were provided. In addition, the case-series design did not allow for the identification of the pathways leading to the drop in CFR.

Orach *et al*^32^ compared the cost and coverage of reproductive health services for refugees and host populations in three rural districts in Uganda using a cross-sectional study with an economic evaluation component. They found that per capita health expenditure was 2.7 times higher for refugees (US$13.12 compared to US$4.85) compared to the host population. Interventions for major obstetric emergencies were also more common among refugees than host populations (1.02%; 95% CI 0.79 to 1.25 vs 0.85; 95% CI 0.80 to 0.90; p<0.05). Cost differences were partly explained by the higher qualifications of staff serving refugee populations. Additionally, medicines, health supplies and laboratory testing and equipment were all more readily available and used more in healthcare settings serving refugees than the general population.

Two studies were identified that evaluated the impact of CHWs on reproductive and maternal health services.^22^ ^23^ Using data from the 2006 Afghanistan Health Survey, a population-based survey, Viswanathan *et al*^23^ examined if the presence of a CHW increased the use of modern methods of family planning, attendance at antenatal care and skilled attendance at birth, all of which form part of the basic package of health services intended to deliver cost-effective primary healthcare in rural areas. When adjusting for individual-level and community-level factors, the presence of a female CHW was significantly associated with increased use of modern family planning (OR 1.61; 95% CI 1.21 to 2.15), antenatal care (OR 2.71; 95% CI 1.87 to 3.92), and skilled birth attendance (OR 1.75; 95% CI 1.18 to 2.58). The authors found no significant difference between having no CHW in the community and having a male CHW.

Mullany *et al*^22^ also found positive health outcomes associated with the implementation of a three-tiered health worker system which included trained birth attendants (TBA) undertaking antenatal care (ANC) and uncomplicated deliveries and health workers who, in addition to the responsibilities of the TBAs, distributed family planning and could administer misoprostol and antibiotics. The third tier consisted of maternal health workers who oversaw complicated deliveries and provided comprehensive emergency obstetric services, including blood transfusions. Compared to baseline, women in the study area were 1.83 times more likely (PRR) to receive ANC (95% CI 1.64 to 2.04) and 1.88 times more likely to use modern family planning (95% CI 1.63 to 2.17). Birth attendance with someone trained in emergency obstetric care increased from 5.1% to 48.7% (PRR 9.55; 95% CI 7.21 to 12.64).

## Quality of reporting in studies

Of the 15 studies, 14 were assessed for quality of reporting using the STROBE checklist and the only randomised controlled trial (RCT)^15^ was assessed using CONSORT. Of the observational studies, three were found to be of low quality,^26^ ^30^ ^31^ seven moderate,^19^ ^20^ ^24^ ^27^ ^29^ ^32^ ^33^ and the remaining four high.^21–23^ ^25^ The RCT was found to be of high quality.^28^

The STROBE assessment allowed specific analysis of common areas of low-quality reporting. For these studies, three broad themes emerged: confounding, bias and appropriately reporting findings. Confounding factors were, on the whole, not clearly described or discussed. Only two studies attempted a control,^21^ ^23^ which is largely unsurprising in the context of observational studies, but other quality aspects addressed by STROBE were also consistently lacking. Particularly, only nine of the selected papers gave a clear account of outcomes, exposures, effect modifiers and confounders under assessment.^21–23^ ^25^ ^27–29^ ^31^ ^33^ This persisted in the low reporting of statistical methods to address confounding, which was only adequate in seven studies.^20^ ^21^ ^23–25^ ^28^ ^29^ For the specific STROBE criteria, only three papers adequately reported efforts used to control bias.^20^ ^22^ ^24^ The majority of studies did not justify sample size, with only 3 of the 15 describing sample size calculations.^21^ ^22^ ^25^ An important theme was identified throughout the studies in the way missing data were handled. Two studies reported how missing data were addressed statistically,^23^ ^27^ with three reporting missing data for variables of interest.^22^ ^23^ ^33^ Four studies^19^ ^22^ ^27^ ^33^ adequately reported non-participation at each stage, although none used a flow diagram to represent numbers of participants. While most of the studies^19–25^ ^27^ ^31^ ^32^ discussed limitations appropriately, one of those that did not also was deemed to overstate the causative attribution of its findings.^30^ Two others^31^ ^22^ were also deemed to overstate their findings. The RCT^28^ was scored 28 of 35 applicable points under the CONSORT quality reporting criteria: the main methodological issue was a lack of any detailed reporting of the randomisation process, although this study was considered high quality as compared proportionally to the STROBE results. Overall, there was a pattern of claiming effectiveness and causation where the study design precludes any such inference.

## Discussion

The review identified only 15 studies on the effectiveness of specific SRH interventions in humanitarian settings that met the inclusion criteria. Interventions resulting in improved health outcomes included the use of impregnated bed nets for pregnant women,^28^ subsidised healthcare for refugees,^21^ female CHWs and tiered community reproductive health service provision.^22^ ^23^ Lower quality evidence was found to support HIV and STI education and condom distribution campaigns,^26^ wider literacy and education programmes,^19^ ^25^ well-implemented birth preparedness interventions and various capacity building initiatives.^20^ ^27^ ^29^ ^33^ No studies were identified that measured the effectiveness of GBV interventions (eg, prevention, knowledge, attitudes).

Other significant gaps included that few studies looked directly at provision of or access to family planning. Many of the interventions were based on education in some format. While undoubtedly a core part of programming in these settings, relatively few studies looked at, for example, scaling up service delivery and only one study conducted an economic evaluation.^32^ This is particularly important, as the context, including existing healthcare delivery system and cultural norms, will likely impact on its ability to improve health. Examination of broader contextual influences, such as interaction between refugee and host populations and the different services available to those groups was deemed beyond the scope of this paper but would be useful in future reviews.

The strength of the evidence identified for this review was variable but generally low. Only one of the papers was able to demonstrate a causative association through a randomised controlled trial,^28^ and only two others adequately attempted comparison with a control group.^21^ ^23^ The vast majority of studies were of preintervention and postintervention case-series design. This is particularly significant, given the strength of conclusions and causative attributions offered by some study authors. The common areas of poor reporting quality identified by the STROBE assessment highlight fundamental methodological issues with most of the available evidence, notably a lack of controlled studies, limited appreciation of clear exposures and confounders and inadequate handling of bias. Most of the studies adequately acknowledged these limitations, but at least four were deemed to have overstated the ability of the study to demonstrate causation, rather than simply observed association.^20^ ^22^ ^30^ ^31^ Statistical analysis was adequate where used. The fact that one-third of studies did not attempt statistical analysis demonstrates a general lack of strong evidence in this field. A further area of concern is a lack of cost-effectiveness analysis within the literature, with only one such study was identified for inclusion in this review.^32^ This highlights major gaps in economic data to inform decision-making for delivering and scaling up SRH interventions.

What may explain this limited evidence? First, there are clearly many logistical challenges to conducting research in humanitarian settings given the high levels of insecurity, scarcity of resources, and population movements. However, higher quantity and quality of research has been conducted in such settings for other health outcomes such as mental health and communicable diseases.^11^ Second, many of the SRH interventions commonly used in humanitarian settings have been evaluated in more stable settings and plausibly assumed to be effective in diverse settings. For example, there is no reason to expect decreased efficacy of injectable contraceptives in a humanitarian setting than in a non-humanitarian setting, but challenges in distribution warrant research on effective delivery mechanisms. Third, there are significant challenges inherent in the use of outcomes such as maternal mortality. This is partly due to the issue of measuring a relatively rare event over an adequate time period, and also attributing changes in maternal mortality to a specific intervention with the use of observational data. The use of well-designed RCTs may address this but is clearly challenging in such contexts. Fourth, proxy measures may be reliably used for certain outcomes such as emergency obstetric care for maternal mortality.^34^ However, despite the use of evidence from stable settings and proxy measures, there remains a need for context-specific evidence from humanitarian settings on the most effective and cost-effective way to deliver SRH interventions. Further research is needed to examine alternative means of communicating behavioural change messages and ways in which existing interventions can be expanded to integrate additional services. The lack of evidence on SRH interventions in humanitarian contexts suggests weaknesses in investigating and rigorously documenting the most effective and cost-effective ways of delivering SRH services and a reliance on the status quo.

This systematic review has a number of limitations. To be included in this study, papers needed to fit within relatively narrow criteria. The strict study design criteria excluded qualitative studies which form a rich part of the literature in this field. However, by including only studies demonstrating quantitative change over time, we expected to derive a specific level of evidence to inform our conclusions. Additionally, only papers written in English and French were included. Owing to the disparate intervention types, indicators and methods used in the included studies, we were unable to conduct a meta-analysis. The STROBE and CONSORT checklists were used to assess the quality of reporting in studies, but more specialist quality assessment tools such as the Cochrane Collaboration's tool for assessing risk of bias and the Newcastle Ottawa Scale would have provided more in-depth reviews of quality.^35^

## Conclusion

This review found some evidence to support increased access and demand creation for family planning services through CHWs, healthcare subsidies and discussions within literacy groups. Education-based interventions also suggest positive results relating to HIV-avoidant behaviours. Involving communities in maternal and child health and birth preparedness programmes, as well as refurbishing clinics and hospital facilities, was also associated with increased positive health outcomes. However, the types of study design, limited use of statistical data, and weak quality mean caution needs to be exercised when interpreting results. While interventions proven efficacious in stable settings are being used in humanitarian efforts, more evidence is required to demonstrate the effectiveness of delivering and scaling-up such interventions in humanitarian crises.

## References

[1] Austin J, Guy S, Lee-Jones L Reproductive health: a right for refugees and internally displaced persons. Reprod Health Matters 2008;16:10–21. 10.1016/S0968-8080(08)31351-2 18513603

[2] McGinn T, Casey S, Purdin S Reproductive health for conflict-affected people: policies research and programmes. London, UK: Overseas Development Institute, 2004.

[3] McGinn T, Purdin S Editorial: reproductive health and conflict: looking back and moving ahead. Disasters 2004;28:235–8. 10.1111/j.0361-3666.2004.00255.x 15344938

[4] Krause S, Bader L *We want birth control: Reproductive health findings in northern Uganda*. New York: Women's Commission for Refugee Women and Children and UNFPA, 2005.

[5] Bosmans M, Cikuru MN, Claeys P Where have all the condoms gone in adolescent programmes in the Democratic Republic of Congo. Reprod Health Matters 2006;14:80–8. 10.1016/S0968-8080(06)28258-2 17101425

[6] Patel P, Roberts B, Guy S Tracking official development assistance for reproductive health in conflict-affected countries. PLoS Med 2009;6:e1000090 10.1371/journal.pmed.1000090 19513098 PMC2682761

[7] McIntosh CA, Finkle JL The Cairo conference on population and development: a new paradigm? Popul Dev Rev 1995;21:223–60.

[8] UNFPA. *International Conference on Population and Development—ICPD—Programme of Action* UNFPA, 1995.

[9] Inter-agency Working Group on Reproductive Health in Crises. Inter-agency field manual on reproductive health in humanitarian settings: 2010 revision for field review. New York: Inter-agency Working Group on Reproductive Health in Crises, 2010. 26203479

[10] World Health Organization. Guidelines and RHL guideline appraisals. Secondary Guidelines and RHL guideline appraisals. http://apps.who.int/rhl/guidelines/en/index.html

[11] Blanchet K, Sistenich V, Ramesh A An evidence review of research on health interventions in humanitarian crises. Cardiff, UK: Enhancing Learning and Research for Humanitarian Assistance (ELRHA), 2013.

[12] Moher D, Liberati A, Tetzlaff J Preferred reporting items for systematic reviews and meta-analyses: the PRISMA statement. Ann Intern Med 2009;151:264–9. 10.7326/0003-4819-151-4-200908180-00135 19622511

[13] Report of the International Conference on Population and Development. *International Conference on Population and Development*; New York: United Nations, 1994.

[14] United Nations High Commissioner for Refugees WHO, United Nations Population Fund, *Reproductive health in refugee situations an inter-agency field manual*. Geneva: UNHCR, 1999.

[15] Nyamtema AS, Urassa DP, van Roosmalen J Maternal health interventions in resource limited countries: a systematic review of packages, impacts and factors for change. BMC Pregnancy Childbirth 2011;11:30 10.1186/1471-2393-11-30 21496315 PMC3090370

[16] Organization WH Essential interventions, commodities and guidelines for reproductive, maternal, newborn and child health. Geneva: WHO, 2011.

[17] von Elm E, Altman DG, Egger M The Strengthening the Reporting of Observational Studies in Epidemiology (STROBE) statement: guidelines for reporting observational studies. Prev Med 2007;45:247–51. 10.1016/j.ypmed.2007.08.012 17950122

[18] Rennie D CONSORT revised—improving the reporting of randomized trials. JAMA 2001;285:2006–7. 10.1001/jama.285.15.2006 11308440

[19] McGinn T, Allen K Improving refugees’ reproductive health through literacy in Guinea. Glob Public Health 2006;1:229–48. 10.1080/17441690600680002 19153909

[20] Casey SE, McNab SE, Tanton C Availability of long-acting and permanent family-planning methods leads to increase in use in conflict-affected northern Uganda: evidence from cross-sectional baseline and endline cluster surveys. Glob Public Health 2013;8:284–97. 10.1080/17441692.2012.758302 23305269 PMC3613974

[21] Raheel H, Karim MS, Saleem S Knowledge, attitudes and practices of contraception among Afghan Refugee Women in Pakistan: a cross-sectional study. PLoS ONE 2012;7:e48760 10.1371/journal.pone.0048760 23133658 PMC3487847

[22] Mullany LC, Lee TJ, Yone L Impact of community-based maternal health workers on coverage of essential maternal health interventions among internally displaced communities in Eastern Burma: the MOM project. PLoS Med 2010;7:e1000317 10.1371/journal.pmed.1000317 20689805 PMC2914639

[23] Viswanathan K, Hansen PM, Rahman MH Can community health workers increase coverage of reproductive health services? J Epidemiol Community Health 2012;66:894–900. 10.1136/jech-2011-200275 22068027

[24] Larsen MM, Sartie MT, Musa T Changes in HIV/AIDS/STI knowledge, attitudes and practices among commercial sex workers and military forces in Port Loko, Sierra Leone. Disasters 2004;28:239–54. 10.1111/j.0361-3666.2004.00256.x 15344939

[25] Woodward A, Howard N, Souare Y Reproductive health for refugees by refugees in Guinea IV: peer education and HIV knowledge, attitudes, and reported practices. Confl Health 2011;5:1–10. 10.1186/1752-1505-5-1 21722361 PMC3152884

[26] Mayaud P The challenge of sexually transmitted infections control for HIV prevention in refugee settings: Rwandan refugees in Tanzania. Trans R Soc Trop Med Hyg 2001;95:121–4. 10.1016/S0035-9203(01)90131-2 11355538

[27] Reid T, van Engelgem I, Telfer B Providing HIV care in the aftermath of Kenya's post-election violence Medecins Sans Frontieres’ lessons learned January–March. Confl Health 2008;2:15 10.1186/1752-1505-2-15 19055803 PMC2634762

[28] Dolan G, ter Kuile FO, Jacoutot V Bed nets for the prevention of malaria and anaemia in pregnancy. Trans R Soc Trop Med Hyg 1993;87:620–6. 10.1016/0035-9203(93)90262-O 8296357

[29] McPherson RA, Khadka N, Moore JM Are birth-preparedness programmes effective? Results from a field trial in Siraha district, Nepal. J Health Popul Nutr 2006;24:479. 17591345 PMC3001152

[30] Purdin S, Khan T, Saucier R Reducing maternal mortality among Afghan refugees in Pakistan. Int J Gynaecol Obstet 2009;105:82–5. 10.1016/j.ijgo.2008.12.021 19232603

[31] Leigh B, Kandeh HB, Kanu MS Improving emergency obstetric care at a district hospital, Makeni, Sierra Leone. The Freetown/Makeni PMM Team. Int J Gynaecol Obstet 1997;59 (Suppl 2):S55–65. 10.1016/S0020-7292(97)00148-3 9389614

[32] Orach CG, Dubourg D, De Brouwere V Costs and coverage of reproductive health interventions in three rural refugee-affected districts, Uganda. Trop Med Int Health 2007;12:459–69. 10.1111/j.1365-3156.2006.01788.x 17313517

[33] Samai O, Sengeh P Facilitating emergency obstetric care through transportation and communication, Bo, Sierra Leone. The Bo PMM Team. Int J Gynaecol Obstet 1997;59:S157–S64. 10.1016/S0020-7292(97)00161-6 9389627

[34] WHO. Monitoring emergency obstetric care: a handbook. Geneva: World Health Organization, 2009.

[35] da Costa BR, Cevallos M, Altman DG Uses and misuses of the STROBE statement: bibliographic study. BMJ Open 2011;1:e000048 10.1136/bmjopen-2010-000048 PMC3191404 22021739

